# ONYX-015: mechanisms of action and clinical potential of a replication-selective adenovirus

**DOI:** 10.1038/sj.bjc.6600006

**Published:** 2002-01-07

**Authors:** S Ries, W M Korn

**Affiliations:** MediGene AG, Lochhamer Strasse 11, 82152 Martinsried, Germany; Division of Gastroenterology and Comprehensive Cancer Center, University of California San Francisco, San Francisco, CA 94132-0128, USA

**Keywords:** ONYX-015, replication-selective adenoviruses, p53

## Abstract

Accumulated knowledge in the molecular processes of tumour development combined with the availability of genetically modified viruses resemble the basis for new promising cancer therapeutics. The main advantages of employing replication-competent viruses are achievement of tumour selective killing and amplification of their oncolytic potential within the tumour mass. In this review, we describe the development of ONYX-015, one of the first and most advanced replication-competent viruses for cancer therapy. We discuss the molecular biology of this therapeutic approach and the interesting results obtained with this virus in clinical trials.

*British Journal of Cancer* (2002) **86**, 5–11. DOI: 10.1038/sj/bjc/6600006 www.bjcancer.com

© 2002 The Cancer Research Campaign

## 

Research on DNA-viruses helped to elucidate basic cellular functions such as DNA replication and gene transcription. These efforts also demonstrated the oncogenic potential of some viruses and led to a detailed understanding of the molecular tools viruses use to achieve their biologic goals. Most fascinatingly, it is evident that many viruses exploit for their replication the same pathways that are altered in cancer cells. Frank McCormick and his group at Onyx Pharmaceuticals were the first to propose taking advantage of this similarity in creating a virus that would selectively replicate in and destroy tumour cells carrying mutations of the *p53* tumour suppressor gene (Bischoff *et al*, 1996). This review summarizes the molecular biologic rationale for the adenovirus mutant dl1520 (now designated ONYX-015 or CI-1042), which has been used to test the possibility of tumour-selective virus replication and it summarizes the ongoing debate over the basic mechanisms involved in replication of ONYX-015. We will provide an up-date on the clinical development of this virus and outline a perspective for its future development.

### Cell cycle control in cancer cells

Tumorigenesis is a multistep process in which mutations in genes involved in cell cycle control and apoptosis accumulate over time (Christofori and Hanahan, 1994; Vogelstein and Kinzler, 1993). Inactivation of tumour suppressors and activation of proto-oncogenes leads to uncontrolled growth and cell division, hallmarks of tumour development. It is now well established that there are common targets of mutation events in cancer development. For example, many tumours have defects in a pathway controlling cell cycle progression that includes cyclin D1, cdk4, p16INK4a, and the retinoblastoma protein (Rb; Figure 1

**Figure 1:**
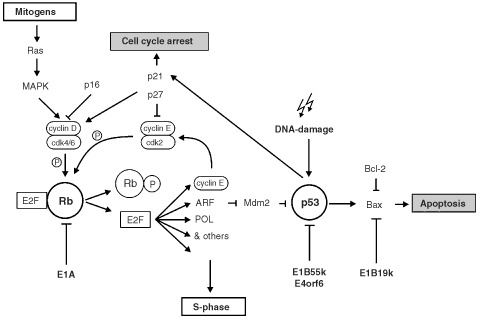
The interaction of adenovirus proteins with the Rb and p53 tumour suppressor pathways. Activating (→) and inactivating effects are indicated (T on side) as well as protein phosphorylation events (P in circle).

; Sherr, 1996). Alterations of this pathway lead to activation of the transcription factor E2F, which promotes cell cycle progression from G1 to S phase. Likewise, most, if not all tumours characterized to date, have defects in the p53 tumour suppressor pathway. Mutations involve either p53 itself or proteins that control its activity or stability such as p14ARF and Mdm2 (Lane, 1999; Levine *et al*, 1991; Steele *et al*, 1998). A variety of therapeutic approaches have been proposed that target these anomalies frequent in cancer and differentiate malignant cells unambiguously from normal cells. The agent discussed here was one of the first to directly target cells bearing mutations in the p53 pathway.

p53 is a transcription factor that can block cell cycle progression and induce apoptosis in response to cellular stress or DNA damaging signals (Giaccia and Kastan, 1998; Levine, 1997). After DNA damage, the p53 protein becomes phosphorylated, is stabilized, and accumulates in the nucleus of the cells (Caspari, 2000; Ljungman, 2000). Phosphorylation leads to conformational changes that protect p53 from degradation via the Mdm2-ubiquitin pathway (Momand *et al*, 1992; Haupt *et al*, 1997). In its role as a transcription factor, p53 binds to the promoters of genes involved in cell cycle arrest and apoptosis, such as p21^WAF1^ and Bax, respectively (el-Deiry *et al*, 1993; Miyashita and Reed, 1995).

The function of p53 is closely linked to the cellular machinery promoting cell cycle progression. This became very clear when a new tumour suppressor protein was identified, p14ARF, which is induced by activation of E2F and acts as an upstream activator of p53 (Bates *et al*, 1998; Palmero *et al*, 1998). p14ARF can bind to Mdm2 and sequester it in the nucleolus of the cell keeping it physically away from p53 thereby preventing p53's degradation (Sherr and Weber, 2000). The result of Mdm2 neutralization by p14ARF is that p53 protein levels will rise and subsequently cause cell cycle arrest and apoptosis. p14ARF also inhibits Mdm2's ubiquitin ligase activity through direct physical interaction (Honda and Yasuda, 1999). Furthermore, recent studies have demonstrated additional mechanisms for p14ARF's function in controlling p53 activity independent of its ability to sequester Mdm2 in the nucleolus (Weber *et al*, 1999). As the cellular p53 protein level rises, p53 binds to the promoter of the Mdm2 gene, stimulating its transcription (p53-Mdm2 negative feedback loop; Piette *et al*, 1997). p14ARF is encoded by the INK4a gene locus, which also contains the gene for the cyclin dependent kinase inhibitor p16Ink4a (Serrano, 1997). p16Ink4a is a component of the Rb pathway and negatively regulates the activity of the cyclinD/cdk4 complexes (Serrano, 1997). Importantly, the INK4a gene locus is frequently targeted by mutations in a variety of human cancers, making it the second most altered gene locus in tumours (Orlow *et al*, 1999; Thompson *et al*, 2001). Amplifications of the Mdm2 oncogene and loss of p14ARF have been reported as an alternative mechanism of inactivating p53-dependent checkpoint control (Cordon-Cardo *et al*, 1994). Defects in both pathways (Rb pathway and p53 pathway) then allow tumour cells to escape protective responses triggered by p53, such as growth arrest and apoptosis, following activation of E2F by oncogenes or DNA-damage.

### Cell cycle control by DNA-viruses

It has been known for many years that a variety of viruses are capable of altering the cell cycle. Viruses have evolved to utilize molecular factors in the infected cell in a way that allows maximizing their replication and production. In this respect, viruses and cancer cells share a common goal: to replicate their DNA freely and efficiently. Not surprisingly, viruses interfere with the same signal transduction pathways that are altered in cancer, promoting G1 to S-phase transition of the cell cycle. In particular, p53 and Rb dependent cell cycle checkpoints are bypassed through virus induced mechanisms.

Viruses have evolved gene products that either interact physically with cell cycle regulatory proteins or activate their transcription, thereby mimicking cell cycle activation in quiescent cells. One major function of the early adenoviral gene products, such as E1A and E1B, is to push the infected cell into the S phase of the cell cycle (Flint and Shenk, 1997), since it is necessary for the virus in order to use the cellular DNA replication machinery to replicate its own genome efficiently. The E1A proteins have been shown to induce quiescent cells to enter S phase by binding to Rb and thereby releasing free E2F (Flint and Shenk, 1997). On the other hand, E1A also induces the expression of p14ARF and subsequent accumulation of active p53 in the nucleus of the cell (de Stanchina *et al*, 1998). This would lead to growth arrest of the infected cell induced via a p21WAF1 triggered mechanism (el-Deiry *et al*, 1993). p53 may also induce Bax, leading to cell apoptosis before the viral life cycle has been completed (Miyashita and Reed, 1995). In both cases viral yields would be significantly reduced. To circumvent this problem, adenoviruses encode another set of early genes, the E1B genes (E1B55K and E1B19K); that protect the infected cell from effects triggered by E1A-induced p53 (Kao *et al*, 1990; Yew and Berk, 1992; Yew *et al*, 1994). The E1B19K protein is a functional homologue of the proto-oncogene Bcl-2 and prevents apoptosis (Rao *et al*, 1992; Debbas and White, 1993). The E1B55K gene product is capable of binding p53 and inactivating it (Kao *et al*, 1990). Together with another early viral protein, the E4orf6 protein, E1B55K exports p53 to the cytoplasm and targets it for degradation (Moore *et al*, 1996; Querido *et al*, 1997). Furthermore, at later times in infection, both proteins act in concert to facilitate the transport of viral late mRNAs while inhibiting the transport of most cellular mRNAs (Babiss *et al*, 1985; Halbert *et al*, 1985). It is noteworthy that other DNA viruses, such as SV40 or HPV-16, also make efforts to eliminate p53. (Lane and Crawford, 1979; Linzer and Levine, 1979; zur Hausen, 1991).

## *IN VITRO* AND PRECLINICAL STUDIES

### ONYX-015: selective replication in tumour cells with altered p53 pathway

The striking similarities of tumour cells and adenovirus infected cells gave rise to the concept of using a mutant adenovirus to selectively eliminate tumour cells, which is based on the observation that in both, tumour cells and adenovirus infected cells, p53 – in its role as ‘guardian of the genome’ – is a major target for inactivation. ONYX-015 contains a 827 bp deletion in the E1B region of the viral genome and a point mutation that generates a premature stop codon preventing expression of a truncated form of the E1B55K protein (Barker and Berk, 1987). Theses mutations render ONYX-015 incapable of blocking p53's function. Indeed, dl1520 is incapable of degrading p53, which therefore accumulates in the nucleoplasm after infection of normal (p53^+^) cells. Infection of normal cells with ONYX-015 should evoke a p53 response: either growth arrest or apoptosis, resulting in aberrant viral replication. In contrast, tumour cells that do not possess a functional *p53* gene (p53^−^ cells) should support replication of ONYX-015. Replication of ONYX-015 should therefore be restricted to p53 deficient cells resulting in selective destruction of cancer cells.

### Effects of ONYX-015 on tumour cells *in vitro*

In the first experiments to test this hypothesis, ONYX-015's ability to grow was tested in tumour cell lines of known p53 status (Bischoff *et al*, 1996). These experiments suggested a correlation between p53 status of the tumour cell lines and susceptibility to ONYX-015. Genetic experiments in tumour cell lines provided further evidence for ONYX-015's p53 selectivity. Functionally p53-deficient cell lines were derived from tumour cell lines with wild-type p53 protein (RKO and A2780) by expression of a dominant negative p53 allele. While ONYX-015 replication is not supported in the parental cell lines, the p53 defective derivative was killed at very low multiplicities of infection (MOI; Bischoff *et al*, 1996; Rogulski *et al*, 2000b; Ganly *et al*, 2001). These experiments provide genetic evidence for an important role of p53 in response to virus infection and underscore the necessity for adenovirus to eliminate p53's function in order to replicate efficiently.

In agreement with the initial hypothesis, investigations in normal cells demonstrated that ONYX-015 does not replicate in normal human mammary epithelial cells and microvascular endothelial cells, while wild-type adenovirus is not restricted in its replication in these cells under identical conditions (Heise *et al*, 1997).

Subsequent work by several laboratories, however, found that a variety of tumour cell lines with wild-type p53 allow efficient replication of ONYX-015 (Goodrum and Ornelles, 1998; Rothmann *et al*, 1998; Turnell *et al*, 1999). In a recent study, we were able to explain some of these observations. We found that lack of p14ARF expression in tumours with wild-type p53 disrupts the p53 signalling pathway leading to high and uncontrolled Mdm2 protein activities and, therefore, facilitates ONYX-015 replication (Ries *et al*, 2000). Through deregulation of Mdm2, p53 is inhibited from exerting its protective effects after adenoviral infection. Re-introduction of functional p14ARF into such tumour cells led to induction of p53 activity and prevented replication of ONYX-015, but not wild-type adenovirus. This protective effect of p14ARF is p53 dependent. Re-introduction of p14ARF did not prevent replication of ONYX-015 in an isogenic cell line, in which the wild-type p53 gene has been deleted by homologous recombination (Ries *et al*, 2000). Thus, genetic lesions affecting molecular factors within the p53 pathway other than mutations of p53 itself can render cells permissive for ONYX-015. Finally, it should be noted that E1B55K has functions other than neutralizing p53, such as promoting the export of late viral mRNAs (Babiss *et al*, 1985; Halbert *et al*, 1985). In some cells types, differences of replication efficacy between wild-type adenovirus and ONYX-015 might be explained by these p53-independent functions of E1B55K.

Until recently, the prevailing view was that adenovirus had developed strategies to negate p53-dependent effects in order to circumvent cellular mechanisms for inhibition of viral replication. In recent studies, it was speculated that wild-type p53 might actually be required for efficient adenovirus replication (Dix *et al*, 2000) despite the fact that several cell lines with various mutations in the p53 gene (including homozygous deletions) supported efficient replication of wild-type adenovirus and ONYX-015. The study fails to assess the actual production of a new virus in cell lines with wild-type p53 and did not examine infected cells for evidence of apoptosis. Therefore, the experiments did not distinguish between cell death due to viral replication or apoptosis. The debate on the role of p53 as a regulator of adenovirus replication was extended recently by an interesting study in which researchers used a mutant p53 molecule containing sequences from the p53 homologue p73 to analyze p53 dependent effects on virus replication (Koch *et al*, 2001). According to the authors, this chimeric molecule cannot be degraded by E1B55K while the function of p53 as a transcription factor remains fully preserved. Curiously, this mutant p53 molecule had no inhibitory effect on replication of wild-type adenovirus or ONYX-015 in normal or tumour cells (Koch *et al*, 2001). This study, however, failed to demonstrate the functionality of the p53/p73 chimera convincingly, in particular its responsiveness to oncogene or DNA-damage-induced signals. Their analysis of transcriptional targets of p53 is restricted to p21WAF1. Of particular importance, they do not examine expression of the proapoptotic gene Bax. Experiments in primary cells have shown that the suppression of ONYX-015 replication by p53 in normal cells is preferentially mediated through Bax (L Johnson and C O'Shea, personal communication). The clinical studies also do not support the view that ONYX-015 replicates in normal cells with functional p53. As discussed in the following paragraphs, ONYX-015 has no demonstrable toxicity in animals or humans, and in some clinical studies can be shown to replicate in tumours lacking functional p53.

### Preclinical animal studies

Subsequent to the initial investigations in cell lines, studies in tumour bearing mice were performed to evaluate the anti-tumour efficacy of ONYX-015, dose-limiting toxicities, effects of scheduling, and the possibility of combining this treatment in a meaningful way with other established therapeutic modalities.

Initial experiments in xenograft models of human tumours (harbouring wild-type p53 or mutated p53) demonstrated significant anti-tumour activity of intratumourally administered ONYX-015 in tumours with mutated (Heise *et al*, 1997). In contrast, ONYX-015 injection into tumours derived from wild-type p53 glioblastoma cells had no inhibitory effect on tumour growth. While mutated p53 tumours showed signs of virus replication, as demonstrated by *in situ* hybridization and immunohistochemistry, this was not the case in the wild-type p53 cells (Heise *et al*, 1997). In addition, mixing experiments were performed in which only a fraction of subcutaneously injected cells has been pre-incubated with ONYX-015. As few as 5% of the injected cells needed to be infected to completely abolish tumour growth of cells with mutant p53, whereas no effect on tumour formation by cells with wild-type p53 cells was unchanged (Heise *et al*, 1999a). These data correlate well with *in vitro* studies and demonstrated significant therapeutic efficacy of ONYX-015 *in vivo* with evidence for p53-dependent virus replication.

Subsequent studies examined xenograft tumours derived from a laryngeal cancer cell line, HLaC, that showed an increased survival without achieving complete remissions following ONYX-015 treatment. Although these cells harbour wild-type p53, the pathway is partly inactivated because of a mutation of the p14ARF tumour suppressor gene. Different dosing schedules were tested and showed that virus application for five consecutive days was superior to a one-time application of the same total virus dose (Heise *et al*, 1999a). Furthermore, of particular importance for the subsequent clinical development of ONYX-015, it was noted that efficacy against this tumour was significantly enhanced when the virus was combined with the chemotherapeutic agents 5-FU or cisplatin (Heise *et al*, 1997). Similarly, combining the virus with radiotherapy resulted in enhanced anti-tumour efficacy (Rogulski *et al*, 2000a). It is conceivable that in tumour cells harbouring wild-type p53 the pro-apoptotic effect of chemotherapy and radiotherapy, respectively, is mediated through DNA-damage dependent activation of p53 (a signalling event that occurs independently of p14ARF). It is well established that the adenovirus E1A protein enhances this effect. The data support the use of ONYX-015 in tumours independently of their p53 status if the treatment is combined with chemotherapy.

Experiments in mice bearing bilateral flank xenograft tumours have addressed the clinically relevant question of whether there is systemic spread of virus to distant tumour sites. While intratumoural injection of ONYX-015 resulted in immunohistochemical detection of virus replication in contralateral, non-injected, tumours, growth of these contralateral tumours was not inhibited (Heise *et al*, 1999b). Nevertheless, growth of contralateral new tumours was reduced following virus injection into established tumours. These results implied that systemic virus spread to distant tumours occurs with suppression of microscopic tumours. The number of infected cells at established distant tumours, however, was apparently too low to initiate progressive, tumour-ablative virus replication. As a possible solution to this limitation, intravenous (iv) application of the virus was investigated in subsequent studies in mice with established subcutaneous xenograft tumours derived from a series of human tumour cell lines, i.v. injection of ONYX-015 caused significant growth inhibition and increased survival in all cases (Heise *et al*, 1999b). *In situ* hybridization experiments demonstrated spread of virus replication throughout the tumour, beginning in focal areas near blood vessels (Heise *et al*, 1999b). However, complete remissions occurred in only a small number of tumours. The reduced efficacy of ONYX-015 given systemically could be the result of a number factors including deficient adenovirus receptor expression, extensive removal of virus by hepatic up-take, and effects of the immune system despite the impaired immunity. These issues will be discussed in the context of clinical studies (below). Nevertheless, the anti-tumour efficacy of systemically administered ONYX-015 was dramatically improved when the virus was combined with the chemotherapeutic agent 5-FU. While treatment with ONYX-015 alone induced only one complete remission (CR) and with 5-FU two CR (seven animals per group), six CRs were observed in animals that received the combined treatment (Heise *et al*, 1997).

## CLINICAL EXPERIENCE

### Intratumoural application of ONYX-015

**Figure 2:**
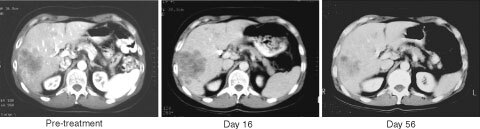
Abdominal CT-scans from a patient with liver metastases from colorectal cancer who received infusions of ONYX-015 into the hepatic artery in combination with 5-FU and Leucovorin (i.v. bolus injection) as part of a phase-II clinical study (Table 1

Based on the encouraging preclinical experience with ONYX-015, clinical trials have been initiated in patients with squamous carcinomas of the head and neck (HNSCC), and pancreatic cancer. Additional studies were performed in patients with premalignant oral dysplasia, ovarian cancer, and liver metastases from colorectal cancer (Figure 2

**Table 1:**
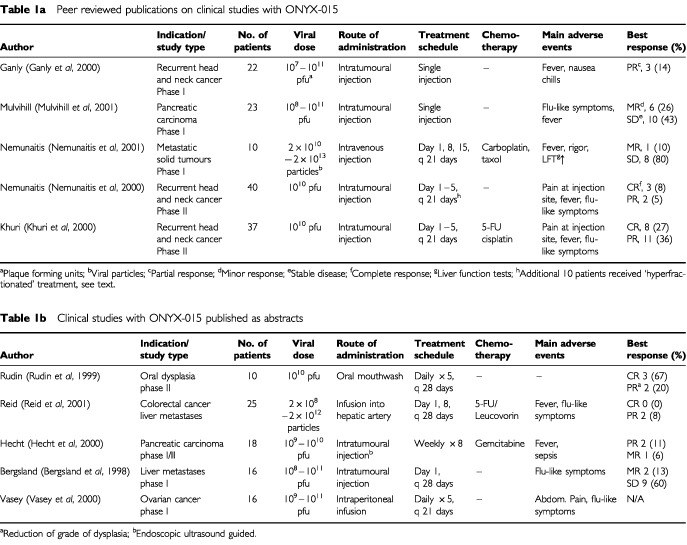
(a) Peer reviewed publications on clinical studies with ONYX-015. (b) Clinical studies with ONYX-015 published as abstracts

). Before enrollment into this study, the patient had experienced disease progression despite treatment with different chemotherapeutic regimens 5-FU/Leucovorin and CPT-11 (Images courtesy of Dr T Reid and Dr D Sze, Stanford University, Palo Alto, CA, USA).

). As these studies have not been published in peer-reviewed journals yet, they will not be discussed in this review.

Phase-I studies involved single intratumoural injections of ONYX-015. In all cases, escalation of the virus dose was possible without that a dose-limiting toxicity was observed and the maximum virus dose that could be administered was based on manufacturing capabilities (Ganly *et al*, 2000; Mulvihill *et al*, 2001). The spectrum of side effects was similar: Most frequently observed were fever, nausea, chills, and a flu-like syndrome (Table 1). In addition, patients with HNSCC experienced pain at the injection site. No significant liver toxicity occurred and there were no signs of disseminated intravascular coagulation. Based on these results, intratumoural injection of ONYX-015 appears to be safe.

With regard to the efficacy of ONYX-015 for human tumours, the largest experience to date is in HNSCC. Evaluation of tumour response at the injected tumour sites showed that three of the 22 patients (14%) experienced objective, partial, tumour responses and an additional ten patients (45%, including two minor responses) showed stabilization of their disease. While detection of viral DNA using *in situ* hybridization technology revealed evidence of intratumoural virus replication in four tumour biopsies that all harboured mutant p53, no statistically significant correlation between p53-status and tumour response could be established. It should be noted, however, that these biopsies were obtained on day eight following injection of the virus, demonstrating prolonged intratumoural replication of ONYX-015. In the same study, the humoural immune response to virus injection was assessed. While 59% of the patients had pre-existing neutralizing antibodies to adenovirus, all but one patient developed increased antibody levels after treatment (Ganly *et al*, 2000). However, the presence of pre-existing antibodies did not correlate with tumour response. This finding agrees with the experience using non-replicating adenoviruses for the delivery of p53 into tumour cells, where a correlation between neutralizing antibodies and transgene delivery could not be established (Clayman *et al*, 1998).

A subsequent phase-II study was conducted in patients with recurrent HNSCC. As a consequence of the animal studies described above, which had shown that sequential doses of virus are superior to single applications, two dosing regimens were examined. The therapy consisted either of intratumoural injection for five days (standard regimen) or twice daily for two consecutive weeks (hyperfractionated regimen). Main side effects were mild to moderate fever and pain at the site of injection; the latter was particularly frequent in patients receiving hyper fractionated therapy.

In patients receiving standard therapy, objective responses were observed in 14% (including three out of 40 patients that achieved complete responses), which were significantly correlated with the presence of *p53* gene mutations. In an additional 12 patients (41%) the disease was stabilized. There were no significant differences in therapeutic activity between the treatment schedules. Interestingly, in the first cycle of treatment viral genomes were detected in the peripheral blood of about half the patients 24 h after the last virus administration, a finding that was interpreted as a sign of intratumoural virus replication. The number of patients with detectable virus declined significantly in subsequent cycles. In parallel, serum levels of neutralizing antibodies increased dramatically (mean titer at baseline, 1 : 51; titer after cycle 1, 1 : 11 869). It is most likely that such high titers of neutralizing antibodies will contribute to a rapid clearance of virus particles shed from the tumours into the peripheral blood. To what extent antibodies interfere with intratumoural virus spread is unclear at this time. Nevertheless, this study demonstrated again the favourable toxicity profile of ONYX-015 and showed unambiguously the anti-tumour activity of the virus in a subset of patients, in particular those with a mutated *p53* gene.

As discussed above, preclinical studies suggested a potential synergistic effect of chemotherapy and ONYX-015. Based on these results, a phase-II study was initiated, aimed to evaluate the combination of ONYX-015 and standard, cisplatin-based chemotherapy in patients with recurrent HNSCC. The results mirrored the preclinical data, including the frequent occurrence of complete remissions. Of 30 evaluable patients, 19 (63%) experienced an objective tumour response, including eight (27%) complete responses. Most interestingly, none of the 19 tumours that had shown objective responses progressed during the observation time of this study. In patients with multiple lesions, only the largest and clinically most relevant tumour had been injected, so that the other tumours served as intra-patient controls. Despite the fact that the injected tumours were generally larger, nine of 11 responded, in contrast to three of 11 non-injected tumours. This statistically significant difference represents one of most convincing pieces of evidence for a specific anti-tumour effect of ONYX-015. Encouraged by these results, a clinical phase-III study has been initiated and is ongoing; patients with first relapse of HNSCC are receiving either combined cisplatin-based chemotherapy and intratumourally injected ONYX-015 or standard chemotherapy.

### Systemic application of ONYX-015

Intravenous administration of ONYX-015 has been explored in a pilot study of advanced lung tumours (Nemunaitis *et al*, 2001). In this study, patients received intravenous injections of up to 2×10^13^ viral particles. The main side effects included, again, fever and rigours. In contrast to the previous study, however, a transient, dose-dependent increase of serum aminotransferase was also observed. In the context of recent concerns regarding the safety of adenovirus vectors, it is important to note that these changes were mild (maximal four-fold increase of serum ALT) In one patient, intratumoural virus replication was documented although no objective tumour responses were observed. The reasons for a potential lower efficacy of systemic administration of ONYX-015, relative to tumour injection, will be discussed below.

### Problematic bio-distribution and mechanisms of virus resistance

It is well documented in animal models that hepatic uptake of systemically injected adenovirus is efficient, reducing the number of infectious virus particles significantly that may reach the target tumour (Fechner *et al*, 1999). Another factor is the frequent presence of pre-existing neutralizing antibodies against adenovirus. In the clinical studies with ONYX-015, the majority of patients presented with neutralizing antibodies and almost all showed a dramatic increase in antibody following the first virus injection. It has been demonstrated that anti-adenovirus antibodies are important inhibitors of antitumour activity of systemically administered adenoviruses and it has been proposed to use methods such as immunoapheresis to remove these antibodies from the circulation before initiation of treatment (Chen *et al*, 2000). Our knowledge about possible mechanisms that are contributing to resistance of target cells against virus infection is still limited. Nevertheless, it is evident that adenovirus-based therapies depend critically on the ability of the virus to enter target cells, a process for which the recently identified Coxsackie- and Adenovirus Receptor (CAR) is most important as it mediates the attachment of the adenovirus fiber protein to the cell surface (Bergelson *et al*, 1997). Recent reports as well as our own experience indicate that CAR expression is frequently lost or reduced in highly malignant tumours (Li *et al*, 1999). Taken together, these mechanisms potentially diminish the amount of virus available to target cells, notably in the context of systemically administered virus. In addition, intercellular barriers, including tissue stroma (containing collagen fibers and CAR-negative fibroblasts) and necrotic areas within the tumour, could limit the spread of ONYX-015 within a tumour. At this point it is unclear, whether pro-inflammatory cytokines, including IL-2 and TNF are inhibiting or promoting viral spread. Equally uncertain is the contribution of T cells. In addition to the deletion of the *E1B55k* gene ONYX-015 harbours a deletion of the E3B region, which contains the gp19k gene that is able to inhibit MHC class-I antigen presentation at the cell surface. It is therefore conceivable that the recognition of adenovirus antigens leads to elimination of infected cells before production of ONYX-015 is sufficient for successfully infecting neighboring cells. However, it is also possible that the co-presentation of tumour- and viral antigens triggers an immune response against tumour cells, in which case ONYX-015 would promote vaccination against tumour antigens. These questions are a subject of current studies. Nevertheless, mathematical models of virus replication in an immune-competent host suggest that the immune-response to ONYX-015 could play a decisive role for the success of this treatment (Wodarz, 2001). This is supported by recent studies illustrating a balance between growth of xenograft tumours in mice and replication of wild-type adenovirus, resulting in detection of replicating virus 100 days following infection without reduction of the tumour mass (Harrison *et al*, 2001).

### Future perspectives

The results from the clinical phase-III study in HNSCC will be available in the first half of the year 2002 and will have a crucial impact on the further development of this therapeutic approach. In addition, future studies of ONYX-015 might include new strategies to overcome the limitations described above. These include the design of replication-restricted viruses with altered receptor specificity, capable of circumventing the problem of loss of expression of adenovirus receptors on target cells, in particular CAR, by utilizing other, cancer specific proteins as alternate receptors. We are currently investigating the possibility of pharmacological restoration of CAR expression at the surface of cancer cells (Anders *et al*, manuscript in preparation). To overcome the need to infect all tumour cells to achieve complete remission, new versions of ONYX-015 have been created that deliver pro-drug converting enzymes to tumour cells, which create high local concentrations of cytotoxic compounds following systemic administration of non-toxic substrates. For example, an E1B55K-deleted adenovirus has been devised, expressing herpes simplex virus-thymidine kinase (HCV-TK), which catalyzes phosphorylation of gancyclovir resulting in highly cytotoxic metabolites (Wildner and Morris, 2000).

ONYX-015 is an interesting new agent for the treatment of solid tumours that shows unambiguous evidence of antitumour activity in a broader range of tumours than initially anticipated. However, the biological mechanisms defining the interaction of this virus with its human host need further exploration. Based on this knowledge, strategies can be developed to overcome problems of bio-distribution and resistance, and, ultimately, to increase the efficacy of systemically administered ONYX-015.
